# Investigating the Effects of Transcranial Alternating Current Stimulation on Cortical Oscillations and Network Dynamics

**DOI:** 10.3390/brainsci14080767

**Published:** 2024-07-29

**Authors:** Sandeep Kumar Agnihotri, Jiang Cai

**Affiliations:** Guangdong Institute of Intelligence Science and Technology, Hengqin, Zhuhai 519031, China; caijiang@gdiist.cn

**Keywords:** brain stimulation, tACS, neuronal model, synaptic dynamics

## Abstract

Transcranial electrical brain stimulation techniques like transcranial direct current (tDCS) and transcranial alternating current (tACS) have emerged as potential tools for treating neurological diseases by modulating cortical excitability. These techniques deliver small electric currents to the brain non-invasively through electrodes on the scalp. tDCS uses constant direct current which weakly alters the membrane voltage of cortical neurons, while tACS utilizes alternating current to target and enhance cortical oscillations, though the underlying mechanisms are not fully understood more specifically. To elucidate how tACS perturbs endogenous network dynamics, we simulated spiking neuron network models. We identified distinct roles of the depolarizing and hyperpolarizing phases in driving network activity towards and away from the strong nonlinearity provided by pyramidal neurons. Exploring resonance effects, we found matching tACS frequency to the network’s endogenous resonance frequency creates greater entrainment. Based on this, we developed an algorithm to determine the network’s endogenous frequency, phase, and amplitude, then deliver optimized tACS to entrain network oscillations. Together, these computational results provide mechanistic insight into the effects of tACS on network dynamics and could inform future closed-loop tACS systems that dynamically tune stimulation parameters to ongoing brain activity.

## 1. Introduction

The human brain’s intricate network of neurons exhibits a remarkable capacity for dynamic coordination, a phenomenon underpinned by synchronized electrical activity known as neuronal network oscillations [1,2,3]. These oscillations and neural network dynamics are disrupted in neuropsychiatric diseases, such as schizophrenia, attention deficit hyperactivity disorder (ADHD), and autism spectrum disorders (ASDs), exhibiting aberrations in synchronization and organization of oscillatory cortical activity [4,5]. Cortical neuronal dynamics and alterations in oscillatory dynamics are associated with the severity of neurodevelopmental and cognitive difficulties found in children [5]. These oscillations, occurring across various frequency bands, play a pivotal role in information processing, cognition, and perception [6,7]. Synchronous oscillatory activity in the cortex reflects neuronal integration and is thought to facilitate communication within and between brain regions [8].

In recent years, there has been an increasing appreciation for the pivotal role of developmental trajectories in shaping the physiological and pathophysiological progression of neuronal circuits [9]. Consequently, the concept of early therapeutic intervention during critical developmental periods has garnered significant attention. This approach is seen as a potential means of redirecting pathophysiological developmental trajectories toward more favorable, healthy outcomes [10,11]. However, understanding neural dynamics to modulate them with specific frequencies remains challenging due to the lack of understanding of how neuronal networks respond to applied stimulation.

Understanding the mechanisms governing these oscillations has been a central pursuit in neuroscience, aiming to modulate the network to treat neurological diseases through electric stimulation. Our research aims to elucidate the intricate interplay between external stimulation and endogenous oscillatory processes, shedding light on the mechanisms by which Transcranial Alternating Current Stimulation (t-ACS) modulates cortical networks. The study presents a comprehensive exploration of the impact of t-ACS on cortical network oscillation and dynamics. t-ACS, a non-invasive brain stimulation technique, has emerged as a powerful tool to investigate and modulate neuronal oscillations in vivo [12,13,14]. By delivering external alternating currents to the scalp, t-ACS has the potential to entrain, amplify, or disrupt ongoing brain oscillations, offering unique insights into the causal relationship between oscillatory dynamics and cognitive functions [15,16].

Our study investigates how t-ACS influences oscillatory frequencies, amplitudes, and phase coherence. It has shown potential to modulate endogenous oscillations to induce sleep [17], consolidate brain memory [18], and increase cognitive power. Studies also suggest that t-ACS improves insomnia and increases sleep duration [19,20,21]. Understanding the underlying neurophysiological mechanisms of how t-ACS impacts different frequency bands is critical for various brain functions [22,23]. Considering the potential implications for neurorehabilitation, cognitive enhancement, and clinical interventions, our research aims to provide insights into the application of t-ACS in treating sleep disorders, enhancing cognitive functions, and possibly treating other neuropsychiatric conditions [24,25]. By unraveling the complex interactions between t-ACS and cortical network oscillations, this study contributes to our fundamental understanding of brain function and may have far-reaching implications for various clinical and therapeutic applications.

## 2. Materials and Methods

A computational model was used in this study to simulate the behavior of neurons and check the network’s activity for inducing the cortical oscillations and entrain the frequency with endogenous oscillations. This model also used the closed-loop method to detect the frequency and was activated to synchronize with same phase and amplitude to generate the effect and network of the model, with the expectation that it will work in real-time with sleep behavior data. The next plan is to use the algorithm in humans with real-time collection of data.

### 2.1. Dynamics of Neuron Model

In this study, we employed a computational modeling approach based on the Izhikevich neuron model [26,27]. This model comprises both excitatory pyramidal neurons and inhibitory neurons and was originally designed to investigate cortical behavior. Our adaptation of this model aims to stimulate the neural network while preserving its dynamic properties. The neuronal parameters in our model are consistent with those previously examined by [28] Izhikevich in the context of intrinsic firing within neural networks, mirroring experimental data obtained from in vitro studies [26]. The dynamics of neurons in our computational model are governed by a set of two differential equations. These equations determine the parameters controlling and sustaining network excitability while also updating the membrane potential of the neurons. The membrane potential updates are described by the following equations.

dV/dt = 0.04V^2^ + 5V + 140 − u + Inoise + t-ACS − G_EX_(V − V_AMPA_) − G_IN_(V − V_GABA_) (1)

U′ = u + a (bV − u)(2)

In our computational model, we update the membrane potential (V) to a new value, at intervals of 0.1 ms (dt). The reverse membrane potential for excitatory neurons using amino hydroxy methyl isoxazole propionic acid (AMPA) receptors (V_AMPA_) is set to 0 mV, while for inhibitory neurons utilizing gamma-aminobutyric Acid (GABA) receptors (V_GABA_), it is established at −80 mV, aligning with their respective behaviors. Additionally, a slow recovery variable (u) is updated according to a specific equation. This variable plays a crucial role in activating potassium (K^+^) ionic currents, deactivating sodium (Na^+^) ion currents, and providing negative feedback to the membrane potential (V). The second equation incorporates parameters ‘a’ and ‘b’, which represent variables for the time scale and sensitivity of the recovery variable (u), respectively. Notably, these parameters are configured differently for excitatory and inhibitory neurons. Specifically, ‘a’ is set to 0.02 for excitatory neurons and ranges from 0.1 to 0.02 for inhibitory neurons, while ‘b’ is set to 0.2 for excitatory neurons and varies between 0.2 and 0.25 for inhibitory neurons.

In our model, we introduced two types of current inputs: I (noise) and transcranial alternating/direct current induced by t-ACS/t-DCS, respectively. The noise current (I noise) was applied to the model within the range of 2 to 5 pA, comprising contributions from both excitatory and inhibitory neurons (5 pA for excitatory neurons and 2 pA for inhibitory neurons). On the other hand, t-ACS was simulated with current amplitudes ranging from 0 to 15 pA, varying according to the specific frequency. Furthermore, the model incorporates a condition where, if the membrane potential (V) surpasses +30 mV, it triggers a spike event. In such instances, both the membrane voltage and the recovery variable are reset using the following equation.

If V ≥ 30 mV, then V = c(3)

and

u = u + d(4)

We have defined parameters related to the post-spike reset potential (c) and the recovery variable (d). For excitatory neurons, the after-spike reset potential (c) is set within the range of −65 mV to −50 mV, influenced by the presence of high-threshold K^+^ conductance. In contrast, for inhibitory neurons, this reset potential remains constant at −65 mV. The recovery variable (d) exhibits variation based on neuron type. For excitatory neurons, it fluctuates between 8 and 2, and its dynamics are influenced by a slow–high threshold K^+^ and Na^+^ conductance. In inhibitory neurons, the recovery variable (d) is fixed at a value of 2. In our model, all afferent excitatory conductance contributes to G_EX_, while inhibitory conductance contributes to G_IN_.

### 2.2. Synaptic Connections and Network

The intricate synaptic connections within the brain present a formidable challenge for comprehension in the current era. Therefore, the creation of a computational model offers a straightforward and comprehensible means of studying neuronal behavior. In this model, synapses are structured to transmit conductance originating from pre-synaptic potentials and the exponential decay associated with each connected neuron. The approach employed for conducting and updating synaptic conductance aligns with established principles in the field [29,30,31,32].

G′_EX_ = G_EX_ × e^−Δt/tEX^ (5)

G′_IN_ = G_IN_ × e^−Δt/tIN^ (6)

G′_EX_ and G′_IN_ correspond to the combined conductance of pre-synaptic action potentials from excitatory and inhibitory neurons, respectively. The decay time constants for excitatory and inhibitory neurons, denoted as t_EX_ and t_IN_, have values of 2 ms and 3 ms, respectively. Δt signifies the time elapsed since the occurrence of the most recent pre-synaptic action potential. The connections among excitatory pyramidal neurons, characterized by g_EX-EX_, incorporate a mechanism involving short-term depression. This mechanism is computed as follows.

g_EX-EX_ = d × G_EX-EX_ (7)

The term g_EX-EX_ refers to the connections among excitatory pyramidal neurons. Within this context, ‘d’ represents a synaptic depression variable, with ‘d’ taking on values of 0 when the synapse is fully depressed and 1 when there is no depression. The recovery time for this depression variable, denoted as td, is set to 300 ms. For all connections involving pyramidal excitatory neurons, the depression variable ‘d’ is dynamically updated based on the occurrence of pre-synaptic action potentials in individual neurons.

D′ = d × r(8)

**Figure 1:**
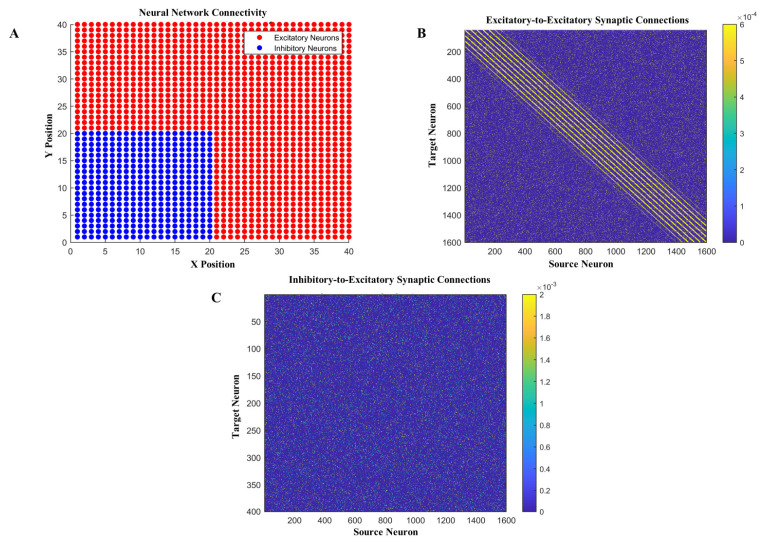
Two-dimensional cortical network of computational model. (**A**) Neural network connectivity between excitatory neurons and inhibitory neurons with excitatory neurons (red) in a 40 × 40 matrix and inhibitory neurons (blue) on the 20 × 20 matrix. (**B**) Connections between excitatory-to-excitatory neurons and their synaptic connections between 1600 neurons. (**C**) Connections between excitatory neurons and inhibitory neurons, where the x axis shows the excitatory neurons and the y axis indicates inhibitory neurons in the computational model.

The parameter ‘r’ represents a depression coefficient, maintaining a constant value of 0.6 throughout. The model comprises 1600 pyramidal excitatory neurons arranged in a 2-dimensional square grid measuring 40 by 40 units and 400 inhibitory neurons organized in a 20- by 20-unit square grid (Figure 1A). The connectivity pattern between pyramidal excitatory neurons and inhibitory neurons reflects sparse local connections. Each pyramidal neuron forms connections with approximately 121 other pyramidal neurons, with a connection probability of 0.0006 (denoted as G_PY-PY_ = 0.0006), a measure implemented to prevent excessive excitation (Figure 1B). The model incorporates synaptic inhibition from both pyramidal excitatory and inhibitory neurons. Each pyramidal neuron connects to around 29 inhibitory neurons, characterized by a connection probability of 0.0002 (G_PY-IN_ = 0.0002). Additionally, each inhibitory neuron establishes connections with approximately 49 pyramidal excitatory neurons, featuring a connection probability of 0.0004 (G_IN-PY_ = 0.0004) on a global random scale (Figure 1C). This configuration contributes to network stability through global negative feedback mechanisms. Within this model, the network exhibits slow rhythmic activity, a phenomenon regulated by a combination of positive and negative feedback mechanisms.

### 2.3. Current-Based Neuromodulation

To introduce a more realistic wave function and variability into the network, we incorporated noise-like current input into the model. A constant current ranging from 2 pA to 5 pA was applied, and at each time step, a random current value was injected into both pyramidal excitatory neurons (ranging from 0 to 5) and inhibitory neurons (ranging from 0 to 2). This approach was aimed at enhancing the model’s realism by introducing stochastic elements. Furthermore, we modulated the tACS current at frequencies between 1 and 9 Hz to investigate its effects at relatively low amplitudes (ranging from 0.00 to 15 pA). These lower amplitudes are more relevant to the study of realistic behavior in the model. In addition, we introduced the t-DCS current into the model to compare its impact on model behavior with t-ACS. This comparison allowed us to assess which cortical brain axis is more effective in influencing neural dynamics. Understanding these dynamics is crucial for evaluating their potential effects on various neural processes, including sleep behavior, which is closely linked to specific neural oscillations in a lower frequency range.

To facilitate this investigation, we developed an algorithm operating in a closed-loop manner. This algorithm detects the model’s frequency, specifically in the small range of frequency (3–10 Hz range). When internal frequency is detected, t-ACS is automatically initiated and synchronized with the model’s frequency, including its amplitude and phase. This synchronization ensures that the model operates similarly, with the forward wave pattern resembling an entrain with the network oscillatory state.

An algorithm, programmed in MATLAB (R2023b licensed version), has been devised to monitor the network’s activity and frequency, Signal Processing Toolbox and custom-based MATLAB script used for analysis of results. This algorithm operates in a closed-loop fashion, triggering the activation of t-ACS and t-DCS at predefined intervals. Simultaneously, it gathers data for analysis, particularly in the form of activity maps, which depict the behavior of both excitatory and inhibitory neurons. These activity maps are normalized according to the total number of neurons and provide a percentage-based representation of individual neuron activity. To visualize the neural network’s activity over time, two-dimensional network algorithms were developed. Our algorithm for detecting the model frequency is a multi-step process designed to capture and analyze the oscillatory patterns inherent in the neural network activity. The neural network activity was recorded over time, resulting in a comprehensive dataset capturing the spiking patterns of individual neurons. We employ a sliding window approach to segment the recorded activity into smaller time windows. This facilitates the analysis of temporal changes in neural activity over the course of the recording. Within each time window, a Fast Fourier Transform (FFT) is applied to convert the neural activity data from the time domain to the frequency domain. The FFT reveals the spectrum of oscillatory patterns present in the neural network during the analyzed time window. The resulting frequency spectrum is examined to identify the dominant frequency component. This is achieved by identifying peaks in the spectrum, which represent the frequency at which the neural network exhibits the most significant oscillatory behavior. Various statistical methods were employed to validate the detected frequency. These include setting a threshold for peak significance, analyzing the signal-to-noise ratio, conducting harmonic analysis, and assessing the consistency of the detected frequency across multiple time windows. The algorithm outputs information regarding the dominant frequency of the neural network activity during each analyzed time window.

The algorithm for monitoring the network’s activity involves recording and analyzing the spiking patterns of individual neurons. The input to the monitoring algorithm is the recorded neural activity dataset, and the output includes details about the firing patterns of neurons over time. The two-dimensional network algorithm involves modeling neural interactions in a spatially distributed manner. Specifically, it incorporates the connectivity and interactions between neurons arranged in a two-dimensional grid. Looking ahead, there are plans to advance these algorithms to enable real-time data analysis in future. This enhanced capability will facilitate the activation of t-ACS during specific sleep stages for a limited duration.

## 3. Results

Our model was meticulously designed with the primary goal of comprehending how t-ACS and t-DCS impact the naturally occurring oscillations within the network when subjected to subtle perturbations. We aimed to investigate both the intricate dynamics of the network and its broader macroscopic behaviors. To evaluate the network’s dynamics comprehensively, we executed computational simulations that encompassed various aspects of neuronal activity. These simulations allowed us to scrutinize the behavior of different types of neurons within the model and their collective interactions.

### 3.1. Dynamics of Synchronized Oscillatory Events in a Computational Cortical Model

**Figure 2:**
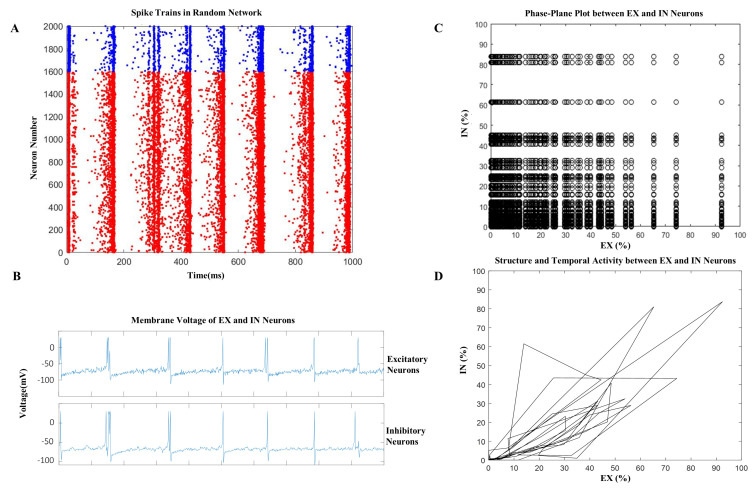
Global oscillatory activity in cortical network of computational model. (**A**) Spike raster plot of network activity with neural spikes, where excitatory and inhibitory neurons synchronize and show network activity (UP states: red (excitatory neurons) and blue (inhibitory neurons) and quiescence (DOWN states: white). (**B**) Membrane voltage traces of excitatory neurons, inhibitory neurons, and spikes of neuros, indicate that neurons activity coordinates with the network. (**C**) Percentage of spiking excitatory and inhibitory neurons during network activity and explicit synchronization. (**D**) Plot showing the temporal activity patterns of EX and IN percentage of neurons over a network activity.

Our computational model was meticulously crafted to investigate the impact of t-ACS and t-DCS, specifically examining their subtle perturbation effects on the microscopic dynamics of a cortical network following stimulation. This model consisted of a two-dimensional network (as depicted in Figure 1A) comprising both excitatory and inhibitory neurons, interconnected through synaptic connections. The network’s activity dynamics were orchestrated by a synchronization mechanism driven by the interplay of excitatory neurons and inhibitory feedback. This orchestration gave rise to transient epochs of active states known as UP-states and periods of inactivity referred to as DOWN-states, with the regulation achieved through the synaptic inhibition exerted by inhibitory neurons. After transitioning from a quiescent DOWN state, the synapses of excitatory neurons rebounded from synaptic depression, facilitating the emergence of the subsequent UP state (Figure 2A, spikes trains plot exhibits the excitatory (red) and inhibitory (blue) neurons) The model’s network activity generated distinct frequencies owing to the loosely coupled interactions between excitatory and inhibitory neurons. Additionally, this activity exhibited synchronization between these two types of neurons (excitatory and inhibitory neurons layers) (Figure 2B) depicted by a phase-plane plot (Figure 2C) with their structural and temporal relationship (Figure 2D). The phase-plane plot illustrates the percentage of spiking excitatory and inhibitory neurons during network activity and explicit synchronization. Figure 2C specifically highlights the dynamic interaction between excitatory and inhibitory neurons, showcasing the proportion of neurons engaged in spiking during network activity. This plot is crucial for understanding the balance and interplay between excitatory and inhibitory forces within the neural network, providing insights into the overall network stability and synchronization patterns. Figure 2D depicts the temporal activity patterns of excitatory and inhibitory neurons as a percentage over the duration of network activity. This temporal analysis allows us to observe the fluctuations in neuronal spiking behavior, offering a detailed view of how excitatory and inhibitory activities evolve over time. By examining these patterns, we can identify periods of heightened synchronization and potential disruptions in network dynamics.

**Figure 3:**
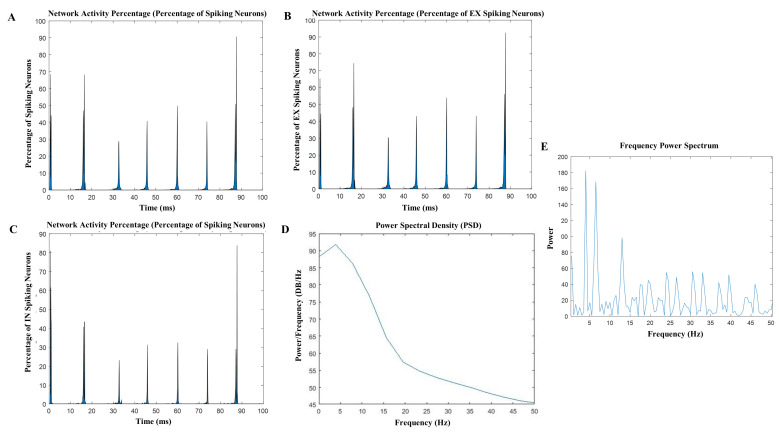
Network activity of excitatory and inhibitory neurons. (**A**) Percentage of excitatory spiking neurons during network activity. (**B**) Percentage of inhibitory neurons during network activity of computational model. (**C**) Percentage of spiking excitatory and inhibitory neurons during network activity and explicit synchronization. (**D**) power spectral density (PSD) graph showing that the maximum frequency is 4 Hz of the network. (**E**) Represents the power/frequency (dB/Hz) against the frequency, where 4 Hz frequency has the maximum power.

**Figure 4:**
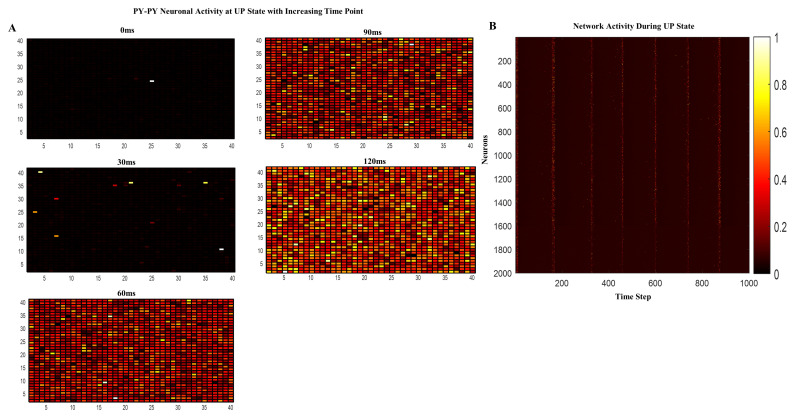
Excitatory neuronal activity during a network UP state. (**A**) Two-dimensional map of excitatory neurons in UP state of network at t = 0, 30, 60,90 and 120 ms, membrane voltage increased with time duration as network activity increased and expanded. (**B**) Spike raster plot maintains the same firings of excitatory and inhibitory neurons in the UP and DOWN state synchronously in the computational model. The neurons fire according to the color code above and are indicated by warmer colors.

Over time, the spatiotemporal patterns of excitation within the network grew, and the model depicted the evolution of activity following the application of t-ACS and t-DCS in both spatial and temporal dimensions. Exploring the model’s rhythmic spatiotemporal dynamics, as depicted in Figure 3A–C, enabled us to achieve a deeper understanding of the mechanisms underlying the impact of t-ACS and t-DCS on network activity and their subsequent influence on other neurons. Additionally, the assessment of power spectral density (PSD) using the Welch technique, as illustrated in Figure 3D,E, provided insights into the power of the endogenous frequency (4 Hz) oscillating within the network. This investigation led to the generation of waveform patterns, enabling us to identify stimulation paradigms based on our newfound mechanistic understanding of t-ACS at the network level, where the hotspot of excitatory neurons (Figure 4A) slowly increased with time and was represented by UP and DOWN states (Figure 4B).

### 3.2. Performance of t-ACS and t-DCS Stimulation on Network Activity

Presently, numerous electrical stimulation devices are employed to modulate the cortex of the brain, specifically to influence the oscillations of the cortical network [33]. Building upon this body of research, we conducted experiments by applying t-ACS and t-DCS stimulation to our computational model, allowing us to make a direct comparison in the context of the cortical network. In our study, both stimulation paradigms were harmonized in terms of amplitude, with both t-ACS and t-DCS set at 5 pA. Furthermore, t-ACS was aligned with the intrinsic frequency of the network, which stood at 4 Hz.

**Figure 5:**
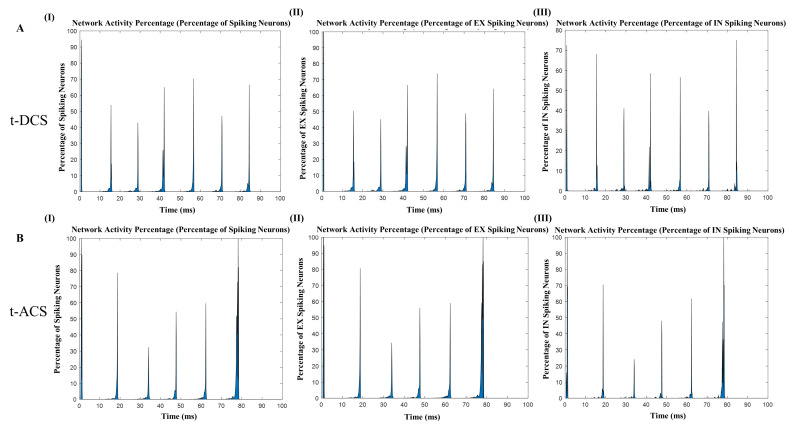
Comparison of tDCS and tACS stimulation. (**A**) Overall network activity of spiking neurons, percentage of excitatory neurons, and percentage of inhibitory neurons when the stimulation of waveform 4 Hz is applied, approximately matched with intrinsic network oscillations with the same amplitude (5 pA), (**B**) t-ACS easily entertained the network and increased the network activity along with the number of excitatory neurons, but tDCS failed to entrain with the network oscillations.

The results obtained from these experiments revealed a noteworthy distinction between the two stimulation techniques. Specifically, t-ACS outperformed t-DCS in terms of its influence on the network’s dynamics. This was evident in the average relative power, where t-ACS exhibited an average relative power of 0.91 at 4 Hz (Figure S2A), whereas t-DCS demonstrated a significantly lower average relative power of 0.33 at the same frequency (Figure S2B). This finding suggests that t-ACS has a more substantial impact on the network’s engagement and synchronization (Figure S1A), particularly at the intrinsic frequency of 4 Hz, compared to t-DCS. Stimulation with t-ACS entrained the network and the percentage of excitatory neurons increased to the maximum, level but for t-DCS, entrainment with the frequency did not demonstrate any effect (Figure 5A(I–III),B(I–III).

To demonstrate that the degree of enhancement in the observed effects relies heavily on the alignment of the t-ACS-induced sine wave’s phase with the endogenously generated network oscillations, we conducted a series of t-ACS simulations. These simulations were characterized by an amplitude of 5 pA, a frequency of 4 Hz, and phase variations spanning from 0 to 2π in increments of 10. The objective was to identify the phase alignment that would yield the most significant impact. The results of these simulations unveiled a pivotal finding: when the phase of t-ACS synchronized with the naturally occurring oscillations within the network, it produced the most substantial effect. This synchronization essentially “entertained” the network’s oscillatory phase and maximized its influence. Additionally, we assessed the activity hotspots within the network involving excitatory neurons at various time points. It became evident that over time, the activity of excitatory neurons displayed a notable increase (Figure 4A). Moreover, these hotspots were primarily generated by the activity of pyramidal-to-pyramidal (PY-PY) neurons. In summation, these findings strongly suggest that t-ACS interacts with the network by aligning with its inherent oscillatory frequency, effectively modulating the spatiotemporal structure of the network. Furthermore, a greater number of hotspots emerged within the network during its UP state in comparison to during t-DCS stimulation.

### 3.3. Recovery of Synaptic Depression in the Network

**Figure 6:**
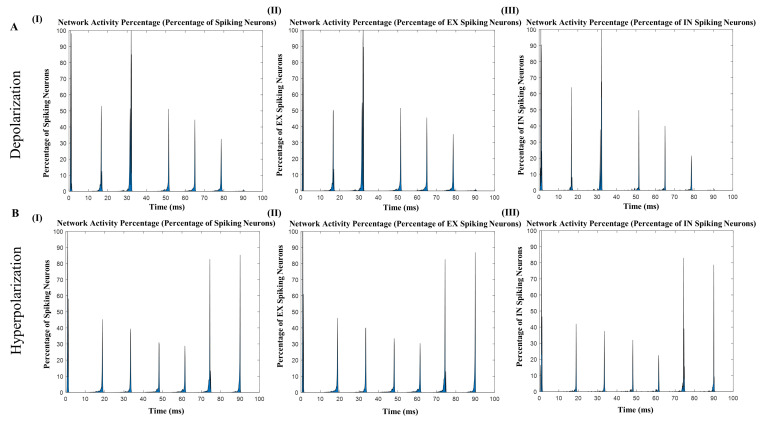
Applying the depolarizing-only stimulation and hyperpolarizing-only stimulation. (**A**) Network activity of spiking neurons, excitatory neurons, and inhibitory neurons after stimulation using only depolarizing-only waveform; networks were entrained with stimulation frequency but were not regular. (**B**) Stimulation of hyperpolarizing-only waveforms exhibits stable entrainment after a few milliseconds with the network and spiking neurons networks and the percentage of excitatory neurons is higher after entrainment.

To investigate the specific influence of individual waveform components, we conducted experiments in which we stimulated only the positive half-wave and negative half-wave of the t-ACS waveform. This allowed us to discern the roles played by positive and negative voltage modulation in t-ACS, which, respectively, generate depolarization and hyperpolarization effects. For these experiments, the amplitude of the unstimulated half-cycle was set to zero, effectively isolating the effects of one half-wave at a time, in comparison to the full waveform. Our findings indicated a significant disparity in the impact of depolarization-only stimulation compared to hyperpolarization-only stimulation. When subjected to depolarization-only stimulation, the network’s activity showed a decline, with both excitatory and inhibitory neuron percentages decreasing (excitatory neurons at 54.44%, inhibitory neurons at 42.8%). However, hyperpolarization-only stimulation presented a different outcome. Network activity initially exhibited a response to hyperpolarization-only stimulation after 75 milliseconds, eventually increasing to a range of 80% to 90% in terms of neuron activity (excitatory neurons at 74%, inhibitory neurons at 62.9%) (Figure 6A(I–III),B(I–III)).

Furthermore, it was observed that depolarization-only stimulation led to reduced oscillation dynamics when compared to full t-ACS stimulation. The average relative power during depolarization-only stimulation was 0.69 at 4 Hz. In contrast, hyperpolarization-only activity exhibited an average relative power of 0.89, which was very close to the values observed during full t-ACS stimulation. In summary, these findings underscore the distinctive roles of depolarization and hyperpolarization in t-ACS and highlight the contrasting effects they exert on network activity. While depolarization-only stimulation led to reduced activity, hyperpolarization-only stimulation demonstrated a capacity to enhance network activity, approaching levels seen during full t-ACS stimulation.

We conducted simulations in the model using the same frequency of 4 Hz and an amplitude of 5 pA but introduced a phase difference. These simulations resulted in a reduction in the percentage of neural activity, particularly a decrease in the percentages of both excitatory and inhibitory spiking neurons. Subsequently, we altered the stimulation frequency to 7 Hz while maintaining the same amplitude. This change in frequency also led to reduced model activity, resulting in decreased percentages of spiking neurons, including both excitatory and inhibitory neurons. Additionally, the average relative power dropped to 0.76. It is worth noting that although the depolarization-only and hyperpolarization-only stimulated networks exhibited a higher degree of regularity (temporal pattern or rhythmicity in the neural spiking activity), it was challenging to discern a clear pattern or progression under depolarizing conditions. To account for the various conditions observed in these experiments, we developed an algorithm capable of identifying the lower frequency range within 1 to 8 Hz for simulating t-ACS stimulation. This algorithm can synchronize with the network, effectively replicating the dynamics seen in sleep paradigms and their associated behaviors.

### 3.4. Resonance-Driven Entrainment

**Figure 7:**
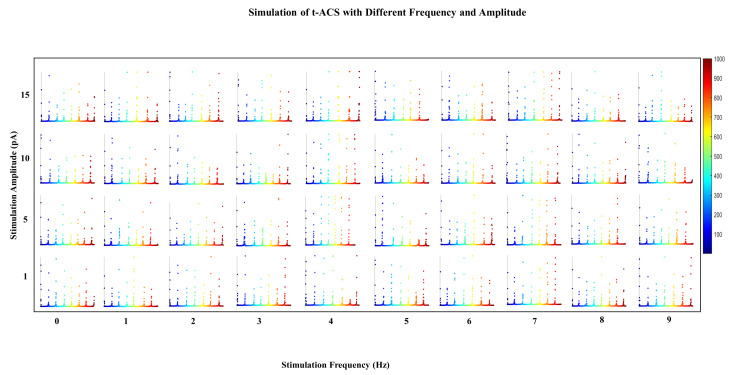
Scatter plot demonstrates a series of t-ACS stimulations, conducted with varying amplitudes (1, 5, 10, and 15) and 0–9 frequencies. The excitatory network’s activity demonstrates resonance with the network under all stimulations. Notably, at lower amplitude (5 pA), resonance occurs at the maximum level with the intrinsic frequency. However, at a higher amplitude, resonance with a 7 Hz frequency is observed. While a range of frequencies entertain the network under all amplitudes, higher amplitudes struggle to maintain sustained entrainment across different frequencies.

We conducted an extensive series of stimulations to assess the extent to which t-ACS can entrain the endogenous oscillations, taking into account various combinations of stimulation amplitude and frequency. Our approach involved applying amplitudes ranging from 1 to 15 pA and frequencies spanning from 0 to 9 Hz. We carefully observed the resulting relative power in the frequency domain, as it provided insights into the network’s response to these stimuli. Within our model, we observed that the most effective entrainment occurred when the applied frequency matched the intrinsic oscillation of 4 Hz, especially at the lowest amplitudes. This condition resulted in both the highest average relative power and amplitude. Additionally, we identified another harmonic frequency at 7 Hz that also exhibited strong entrainment characteristics, with notably high average relative power and amplitude. Conversely, other frequency oscillations had a less pronounced impact on the network when paired with weak amplitudes (Figure 7).

Interestingly, as the stimulation amplitude increased, a wider range of frequencies successfully entrained with the network. This phenomenon can be likened to the concept of the “Arnold tongue”, a frequently observed occurrence in dynamic systems [34], where entrainment is most effective within specific parameter ranges. To quantify the stability of entrainment, we extended our simulations to 5 s of stimulation (Figure S4). Our findings highlighted that the 4 Hz frequency, when paired with lower amplitudes, had a significant and enduring impact, remaining stable over time. In contrast, in all other cases, we observed a more gradual transition between epochs of stable entrainment and periods of desynchronized activity within the network.

### 3.5. Synchronize the t-ACS with Intrinsic Phase, Amplitude, and Frequency

**Figure 8:**
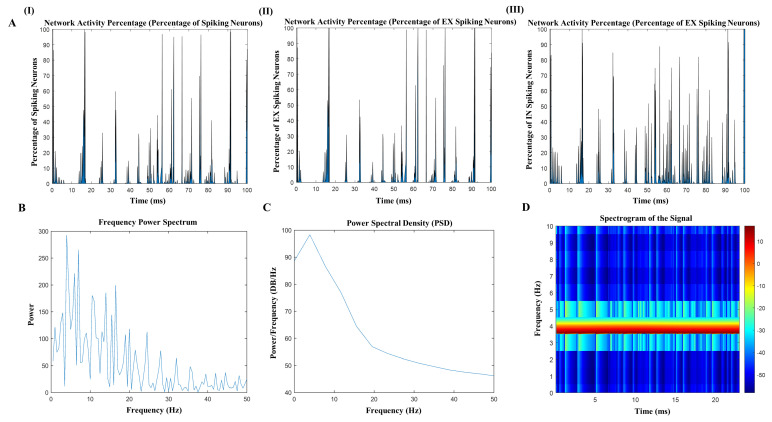
The algorithm is run with same phase, frequency, and amplitude along with the network oscillations. (**A**) algorithm identified the phase, frequency and amplitude of the networks and generate the same waveform to entrain with the network and spiking neurons and excitatory neuron networks with average relative power 0.98. (**B**,**C**) Spectrogram of stimulated frequency matched with the networks oscillations frequency and (**D**) show power of lower frequency band after stimulation with frequency.

As previously observed, variations in frequency, phase, and amplitude exert distinct effects when stimulating the network. These variations play a crucial role in stimulating additional neurons within the network, ultimately resulting in a positive impact on inducing network-level changes in the brain. When we achieved synchronized waveforms characterized by specific phase, amplitude, and frequency parameters, we witnessed a significant influence on the network. Importantly, this influence was achieved without disrupting the network’s inherent dynamical structure, especially within the delta (0.5–4 Hz) and theta (4–8 Hz) frequency ranges. To harness the advantages of synchronization without disrupting the network’s natural dynamics, we developed an algorithm (Figure 8A(I–III)). This algorithm is capable of detecting the intrinsic phase and frequency of the computational model network. Subsequently, it initiates a t-ACS waveform that precisely matches the detected phase and amplitude. This approach allows for seamless entrainment of the t-ACS with the network, effectively aligning the external stimulation with the network’s intrinsic dynamics without causing disruption.

We executed our model in conjunction with the integrated algorithm, and the results were highly efficient. The algorithm successfully synchronized with the intrinsic frequency and phase of the network, achieving the desired amplitude levels. As a result, there was a remarkable increase in the activity of spiking neurons within the network, reaching approximately 93% in terms of both excitatory and inhibitory neuron activity. The alignment of frequency, phase, and amplitude had a notable impact on the network’s dynamics. Initially, it led to an augmentation in the power of the intrinsic frequency, which was quantified by analyzing the power spectral density (PSD) before and after the application of the aligned stimulation. The frequency power spectrum plot, shown in Figure 8B, demonstrates a significant peak at the 4 Hz frequency shortly after alignment, indicating enhanced power in this range. However, as time progressed, we observed a reduction in the presence of alpha, beta, and gamma waves. This reduction is evidenced by continuous PSD analysis, as illustrated in Figure 8C. The plot shows a decline in power within these frequency bands over the duration of the experiment, suggesting a shift in the network’s oscillatory dynamics due to the prolonged effect of the aligned stimulation.

Our algorithm is engineered to detect the dominant frequency within the range of 0.5 Hz to 100 Hz and synchronize the amplitude and phase to harmonize with the cortical network, specifically at positions f3 and f4 (subject to verification) [17]. It is important to note that while these findings are promising, further validation is required in vivo. Additionally, this algorithm holds the potential for diverse applications beyond inducing sleep. It can be adapted for stimulating specific cortical regions of the brain, potentially enhancing cognitive functions and offering a versatile tool for various neurostimulation purposes.

## 4. Discussion

Recent studies in the field of transcranial Alternating Current Stimulation (t-ACS) have shown promising results, particularly in its capacity for precise modulations of cortical stimulation associated with motor and cognitive functions [35]. In some of these studies, t-ACS has been found to effectively induce sleep and enhance memory processes, leading to significant improvements in cognitive functions [17,36]. These findings hold substantial promise for addressing a wide range of sleep and memory-related disorders. By harnessing the potential of t-ACS to improve sleep and memory, there is the possibility of mitigating the impact of conditions such as insomnia [19], sleep apnea [37], memory loss [38], and neurodegenerative diseases like Alzheimer’s [38] and Parkinson’s disease [39]. The application of t-ACS as an intervention strategy offers hope for ameliorating the challenges posed by these conditions and ultimately enhancing overall cognitive well-being.

Brain rhythms, or neural oscillations, play a critical role in various cognitive and physiological processes. Disruptions in these rhythms are associated with several neurological and psychiatric conditions, including Alzheimer’s disease and sleep disorders. Understanding these rhythmic changes is essential for developing targeted interventions. Alzheimer’s disease (AD) is characterized by progressive cognitive decline and memory impairment, with key disruptions in gamma (30–100 Hz) frequency bands [40,41], beta and gamma bands [42], and different brain regions between 8–30 Hz frequency [43]. Treatment of AD has been shown to be effective through flickering light around 40 Hz, which reduces the load of plaques [44]. This suggests that it is possible to treat neurological diseases through frequency modulation. Our research highlights the potential for tACS to target these disrupted rhythms, particularly by modulating gamma activity to improve cognitive functions. Lower brain rhythms also play a role in sleep disorders, including insomnia. These disorders are often linked to alterations in brain rhythms such as heightened beta activity and disruptions in delta and theta bands during sleep. These changes impact the restorative processes during slow-wave sleep (SWS) and contribute to cognitive impairments and mood disturbances [17,19]. Our study suggests that t-ACS, particularly at lower frequencies like 4 Hz, can enhance delta activity and promote synchronous low-frequency oscillations, thereby improving sleep quality and overall cognitive function. Our research aims to explore how t-ACS can modulate these brain rhythms to potentially ameliorate the symptoms associated with these conditions. By targeting specific frequency bands, such as enhancing delta activity for sleep disorders or modulating gamma rhythms in AD, t-ACS holds promise as a therapeutic intervention. However, it is crucial to tailor the stimulation parameters to the unique rhythmic disturbances present in each condition, highlighting the need for individualized treatment approaches.

In the course of our investigation, we employed a computational model to delve into the intricate mechanisms underpinning the modulation of network dynamics by t-ACS. The computational model we utilized comprised 2000 neurons, affording us the capability to explore synchronization within a network that exhibited intrinsic oscillations at 4 Hz, oscillating cohesively across the entire network. Our findings underscore the significant advantages of employing such a model. Notably, we observed the network’s stability in terms of interactions between pyramidal (PY) and inhibitory (IN) neurons, leading to the generation of diverse frequency ranges. Intriguingly, our results indicate that the complex dynamics of the network’s interaction with applied t-ACS can be distilled into a straightforward model driven by periodic forces, offering valuable insights into average relative power and amplitude dynamics. Relevant to our study, Polania [45] conducted research suggesting that a 6 Hz t-ACS frequency, applied to the frontoparietal area, had a substantial impact on memory performance. It is worth noting that these effects were observed to be highly dependent on the phase of the 6 Hz t-ACS, with out-of-phase stimulation resulting in decreased memory performance. These findings collectively emphasize the intricate relationship between t-ACS parameters, network dynamics, and cognitive performance, shedding light on the potential of t-ACS as a modulatory tool in cognitive neuroscience.

This study reinforces the principles inherent in our computational model, underscoring the critical influence of phase and frequency on performance when not in synchronization with resonance dynamics. In our computational framework, the application of t-ACS exerted a notable effect on endogenous oscillations during simulations. This effect was achieved through precise control over the initiation site of oscillations. Subsequently, these oscillations rapidly propagated laterally through local excitatory connections, effectively surpassing a threshold and fully engaging the initial site, maximizing its potential. It is pertinent to note that comparable thresholds and non-linearities are commonly encountered in oscillating neuronal networks. These networks often exhibit a pattern characterized by alternating epochs of quiescence and periods of heightened neuronal firing, reminiscent of cortical slow oscillations [46,47,48]. This study’s findings reaffirm the significance of understanding the interplay between t-ACS parameters and network dynamics, offering valuable insights into the modulation of neuronal oscillations and their potential implications in various cognitive processes.

Transcranial Alternating Current Stimulation (t-ACS) can modulate the excitation-inhibition (E/I) balance in neuronal networks, amplifying t-ACS-induced entrainment. The E/I balance refers to the relative contributions of excitatory and inhibitory synaptic inputs to neuronal events, such as oscillations or sensory responses. When the t-ACS frequency closely matches the endogenous frequency, phase locking between endogenous oscillations and the exogenous electric field is enhanced, thereby amplifying the t-ACS-induced entrainment via E/I balance [49]. The effects of t-ACS on cortical networks likely depend on the E/I balance. Specifically, a particular t-ACS phase increases excitability when cortical activity is excitation-dominated but decreases excitability when it is inhibition-dominated [50,51]. In our study, we modulated t-ACS at frequencies between 1 and 9 Hz and found that a 4 Hz frequency was particularly effective at enhancing intrinsic frequency power. This low-frequency t-ACS promotes synchronization of neuronal firing, enhancing excitatory drive and engaging inhibitory neurons to a lesser extent. In-phase t-ACS reinforces natural network rhythms, increasing excitatory drive, while anti-phase t-ACS can dampen excitatory activity and enhance inhibitory control, which is potentially useful for conditions like epilepsy. We observed that specific t-ACS frequency and phase combinations could either enhance or suppress overall network activity.

Hence, the dynamics unveiled in this investigation possess a broad applicability across diverse network setups and varying frequencies of endogenous oscillations. Our model network demonstrated a remarkable sensitivity to subtle disturbances, effectively mediating entrainment with intrinsic oscillations t-ACS stimulation. The outcomes of our model underscore the pivotal role played by increased connectivity among distributed neurons. This heightened connectivity, especially among highly interconnected nodes, has the capacity to trigger a transition between two markedly distinct activity states: the UP state and the DOWN state. These states represent two contrasting modes of network activity, and the results shed light on the potential for such transitions, even under conditions of weak perturbation. These findings contribute to bolstering the biological plausibility of our model and highlight the significance of subtle perturbations in influencing network behavior and transitions between distinct activity states. Moreover, they underscore the broad applicability of our model’s insights across various network configurations and endogenous oscillation frequencies, augmenting our understanding of network dynamics [52,53].

Our research findings also imply that short-term synaptic depression plays a crucial role in enabling t-ACS to exert a significant impact on cortical oscillations. Specifically, during the hyperpolarizing phase of stimulation, the network was maintained in a manner that facilitated the recovery of synaptic depression in all excitatory synapses. This recovery process, in turn, permitted a high-gain positive feedback loop of excitatory interactions during the UP state of network activity. Consequently, this mechanism contributed to the enhancement of synchronization within the network [54,55]. These insights underscore the intricate dynamics at play in the modulation of cortical oscillations by t-ACS, shedding light on the role of synaptic depression in shaping network behavior and synchronization. Notably, the importance of phase synchronization with the frequency becomes evident, which has significant implications for various applications of deep brain stimulation. Research indicates its crucial role in enhancing visuospatial working memory [56], improving attention [57], and promoting stroke recovery [58] through t-ACS. Moreover, synchronization is a key factor in home-based t-ACS treatments for Alzheimer’s disease [59], memory enhancement [38], and the improvement of motor and cognitive functions in Parkinson’s disease [39].

Our algorithm was meticulously designed to operate in a manner that captures low frequencies, including their corresponding amplitudes and phases. It utilizes this information to precisely synchronize t-ACS with the network’s initial frequency. This approach ensures that the network’s intrinsic dynamics remain undisturbed and seamlessly integrate with the endogenous cortical oscillations. This synchronization strategy stands in stark contrast to our simulated scenarios in which different frequencies and amplitudes were applied, resulting in a lack of stimulation for the endogenous network. Our research outcomes also underscore the superior effectiveness of t-ACS compared to t-DCS. While t-ACS rapidly synchronizes with cortical dynamics during the initial stage, enhancing its efficacy, t-DCS appears less effective in this regard. Furthermore, our findings pertaining to resonance dynamics warrant careful consideration when selecting stimulation frequencies. We observed that cortical oscillations were strongly influenced by stimulation frequencies that fell within the range between the dominant endogenous frequency and its harmonic frequency. These results emphasize the importance of precise frequency selection to avoid perturbing or suppressing critical cortical oscillations [2,60].

A significant implication arising from our findings is the critical importance of matching the frequency of applied stimulation to the frequency of the endogenous oscillatory state. However, when considering macroscopic activity, such as the data assessed by EEG, it typically reveals a different frequency of 1/f spectrum characterized by a lack of a distinct, prominent peak frequency [61,62]. This stands in contrast to our model, where a clear peak frequency was evident. Consequently, the selection of an appropriate stimulation frequency presents a substantial challenge in the absence of a discernible preferred resonance or peak frequency in macroscopic EEG activity. In light of this challenge, our findings and algorithm advocate for the implementation of closed-loop feedback systems. This approach holds promise for future investigations, including animal and human studies, in which the stimulation waveform can be dynamically adjusted to align with ongoing activity patterns. By adopting a closed-loop approach, researchers can adaptively modulate stimulation parameters to better suit the complex and variable nature of macroscopic brain activity, enhancing the precision and effectiveness of neuromodulation techniques. Our study focused on modulating frequencies within the 1–9 Hz range, particularly at 4 Hz, due to its significant impact on network dynamics. We recognize the complexity and diversity of brain rhythms in living organisms, as observed in EEG studies, and our current model, while simplified, serves as a foundational framework. Future work will involve validating our findings with EEG data, incorporating diverse rhythmic patterns, and simulating various physiological and pathological conditions. We aim to enhance the model’s realism and applicability by integrating more detailed synaptic connections and collaborating with experimental researchers to align our simulations with empirical data.

Our research offers a promising avenue for future exploration by expanding simulations to a larger network scale, which will help us to assess scalability and explore brain activity modulation in neurological disorders such as insomnia, dementia, and epilepsy. Integrating experimental data could enhance our approach by refining the model to align more closely with real-world neural dynamics. However, our current approach has limitations. It does not elucidate the mechanisms underlying the sustained effects of t-ACS or the post-stimulation offset, which are crucial for optimizing protocols and predicting long-term outcomes. Additionally, our model lacks mechanisms related to long-term potentiation or plasticity, which are essential for capturing neural adaptations to stimulation. In vivo validation of our findings is also necessary to verify the predictive power of our simulations and translate insights into practical applications. Furthermore, our study focuses on a narrow frequency range (1–9 Hz) and specific cortical areas. Exploring a broader frequency spectrum and different brain regions could provide a more comprehensive understanding of the effects of t-ACS and its potential for diverse therapeutic applications. Addressing these limitations in future research is vital for advancing neuromodulation and developing effective interventions for neurological disorders.

## 5. Conclusions

Our research provides key insights into the intricate dynamics of t-ACS in cortical networks. We found that frequency synchronization between applied stimulation and endogenous oscillations is critical for preserving and enhancing network activity. Our computational modeling highlights the superiority of t-ACS over t-DCS for cortical entrainment during initial stimulation. However, the absence of a clear resonant peak frequency in EEG poses challenges. This advocates for closed-loop systems that dynamically adjust t-ACS parameters to match ongoing brain activity patterns. Such adaptive systems hold promise for optimizing stimulation effects in future animal and human research. Overall, our work elucidates mechanisms of t-ACS neuromodulation, underscoring the potential of closed-loop t-ACS to precisely modulate cortical oscillations. This paves the way for further research on optimizing and applying t-ACS to advance our understanding and treatment of brain disorders.

## Data Availability

Due to institutional regulation and project guidelines, data are available on request to corresponding author and after approval from the project board.

## Supplementary Materials

The following supporting information can be downloaded at https://www.mdpi.com/article/10.3390/brainsci14080767/s1, Supplementary Figure S1: Phase synchronization between 0 and 2 pi with t-ACS and neural network: (A) network activity of spiking neurons represented by blue color and stimulated t-ACS activity represented by red color where mean phase relation across t-ACS and network activity closely around pi (180 degree) which extended to the outermost circle indicates perfect synchronization (yellow circle). (B) Phase synchronization along with different phases between 0 and 2 pi; different phases are shown by yellow lines which indicate the strength of synchronization associated with the corresponding phase difference and at pi (180) strength of synchronization depicted by the length of the yellow line from the center. Supplementary Figure S2: Comparison of relative power induced by t-DCS and t-ACS stimulation: (A) The spectrogram of t-DCS stimulation illustrates a lower power level (average relative power, 0.33). (B) In contrast, t-ACS simulation shows a dramatic increase in average relative power (0.91) after selecting 4 Hz to align with the intrinsic network oscillation frequency. Supplementary Figure S3: Exploration of relative power across varying amplitudes and frequencies: The model simulation incorporates diverse amplitudes (1, 5, 10, and 15) and frequencies ranging from 1 to 9 Hz, representing the dynamic interplay with network oscillations. Relative power, depicted on a color-coded scale from blue to yellow (0 to 1), reveals distinctive patterns. Notably, the 4 Hz frequency exhibits a relatively higher power, synchronizing with the inherent oscillations of the network. Supplementary Figure S4: Dynamic network oscillation activity over a 5 s duration: The model’s 5 s simulation unfolds a captivating narrative of synchronized neuronal firing. During this time span, a detailed examination reveals the percentage of spiking neurons (A), offering insights into the rhythmic patterns of both excitatory (B) and inhibitory neurons (C). This depiction serves as a valuable lens into the model’s sustained behavior over an extended duration, shedding light on the temporal dynamics that shape its response. Supplementary Figure S5: Spike train visualization under varied stimulation conditions: The neural network’s response to different stimulations, specifically t-ACS and t-DCS, has been meticulously captured in this spike trains plot. Neurons subjected to t-ACS are distinguished by the striking hue of red, while those under the influence of t-DCS emanate a warm yellow glow. In contrast, the unstimulated neuron network is represented in a serene blue. This visualization not only provides a nuanced depiction of how different stimulation modalities impact neural firing but also unravels the temporal dynamics, showcasing distinctive patterns during the UP state, where neurons fire in concert, and the DOWN state, characterized by quiescent neurons firing at regular intervals. Supplementary Figure S6: Comparison of mean voltage outputs under varying amplitudes and frequencies. (A) Presents the temporal evolution of mean voltage (ms) following simulation with t-DCS (amplitude 1) and t-ACS (amplitude 1) across frequencies 0–9, including the no-stimulation condition. Notably, the mean voltage for t-DCS exhibits fluctuations between −65 and −40, while for t-ACS, it varies between −70 and −55. (B) Compares mean voltage under 5 different amplitude conditions for both t-DCS and t-ACS across frequencies 0–9, revealing fluctuations ranging from −70 to −57. (C) Explores the divergence between amplitude 10 of t-DCS and t-ACS across frequencies 0–9. (D) Describes the influence on mean voltage after stimulation with t-DCS (amplitude 15) and t-ACS (amplitude 15) across frequencies 0–9, including the no-stimulation scenario (depicted in blue). Supplementary Figure S7: Comparison of average firing rates in the presence of t-DCS and t-ACS, with a reference to the no-stimulation condition: (A) Explores the firing rates for t-DCS across amplitudes 1, 5, 10, and 15, compared with the baseline of no stimulation (depicted in black). (B) Illustrates the variations in average firing rates for t-ACS at amplitude 5 across frequencies 0–9. (C) Demonstrates the collective impact of t-DCS (amplitudes 1, 5, 10, and 15) and t-ACS (amplitude 5) at frequencies ranging from 0 to 9, in comparison to the no-stimulation condition.
