# Supplementary material for: Investigating the Effects of Transcranial Alternating Current Stimulation on Cortical Oscillations and Network Dynamics
Source: Brain Sci. 2024 Jul 29;14(8):767. doi: 10.3390/brainsci14080767 (PMC11353238; doi:10.3390/brainsci14080767)
Supplement: Supplementary file 1 [file brainsci-14-00767-s001.zip › brainsci-3113597-supplementary.pdf]

Figure S1

Phase Synchronization

A

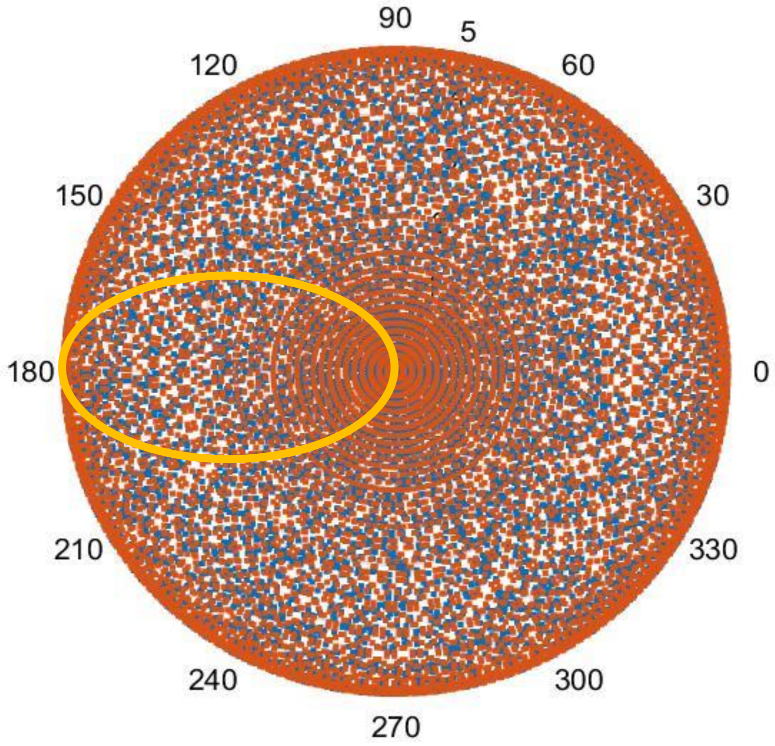

B

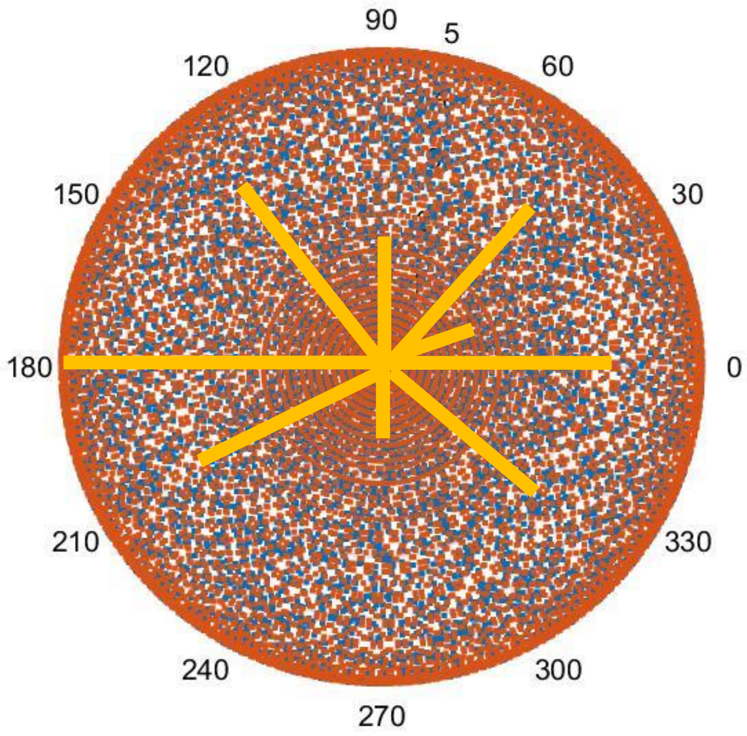

Figure S2

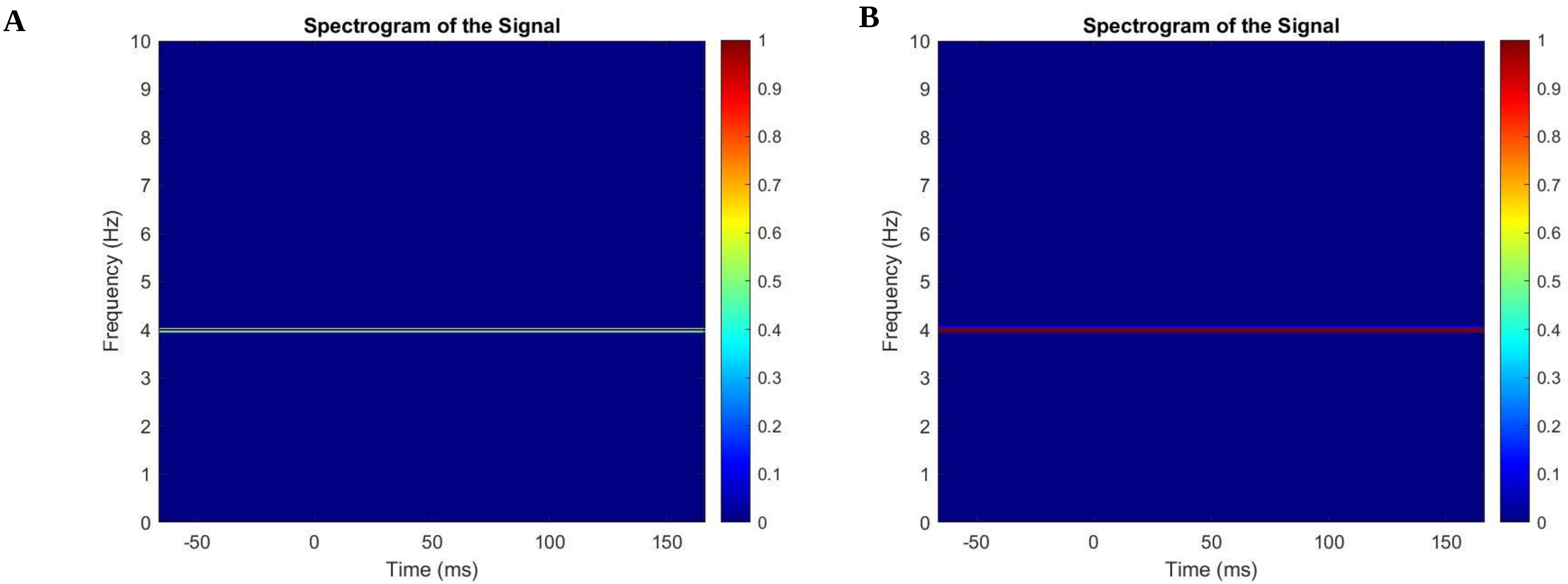

T-DCS with relative power = 0.33

T-ACS with relative power = 0.91

Figure S3

Relative Power Regarding Amplitude

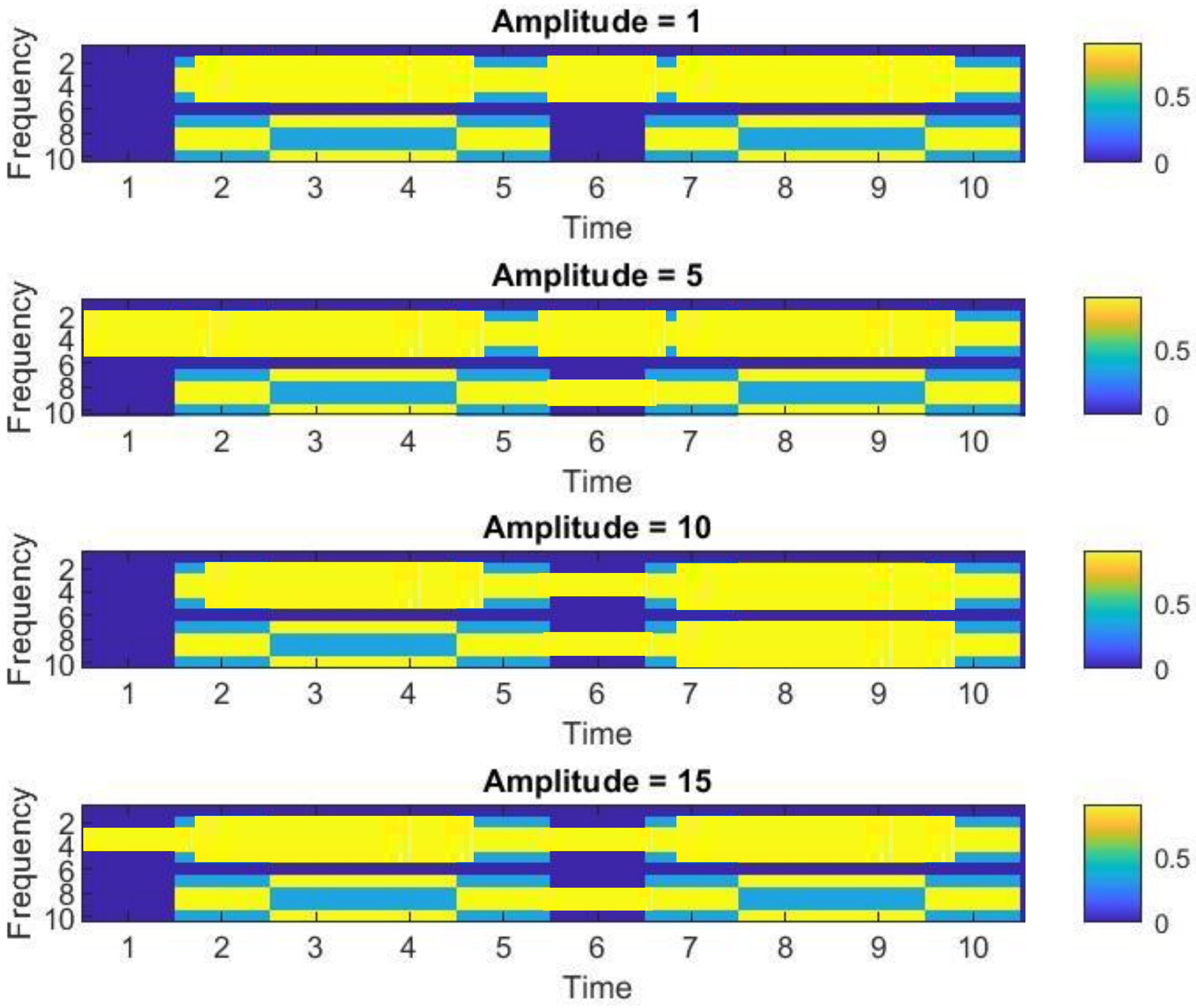

Figure S4

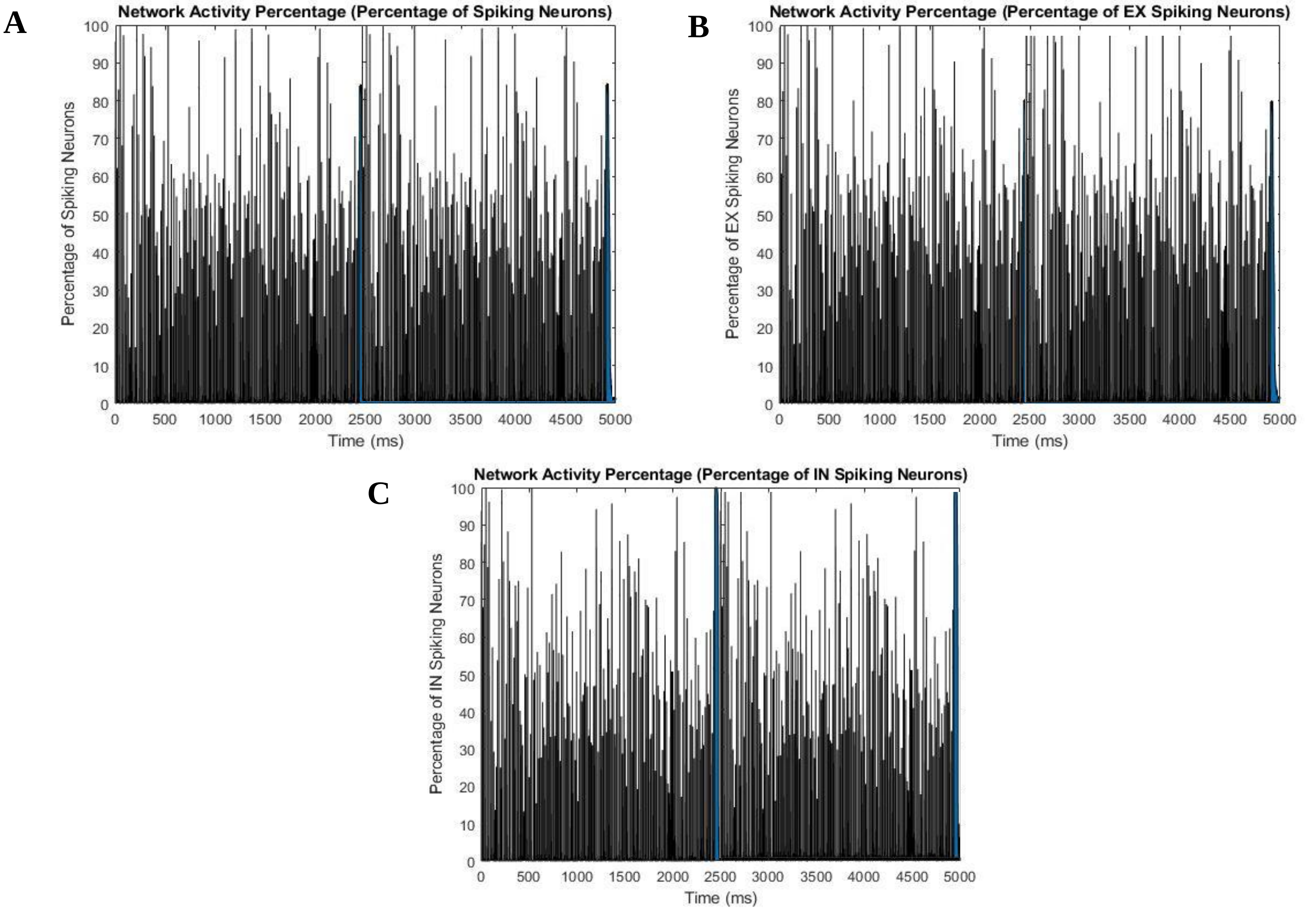
A
Network Activity Percentage (Percentage of Spiking Neurons)
Percentage of Spiking Neurons
Time (ms)
B
Network Activity Percentage (Percentage of EX Spiking Neurons)
Percentage of EX Spiking Neurons
Time (ms)
C
Network Activity Percentage (Percentage of IN Spiking Neurons)
Percentage of IN Spiking Neurons
Time (ms)

Figure S5

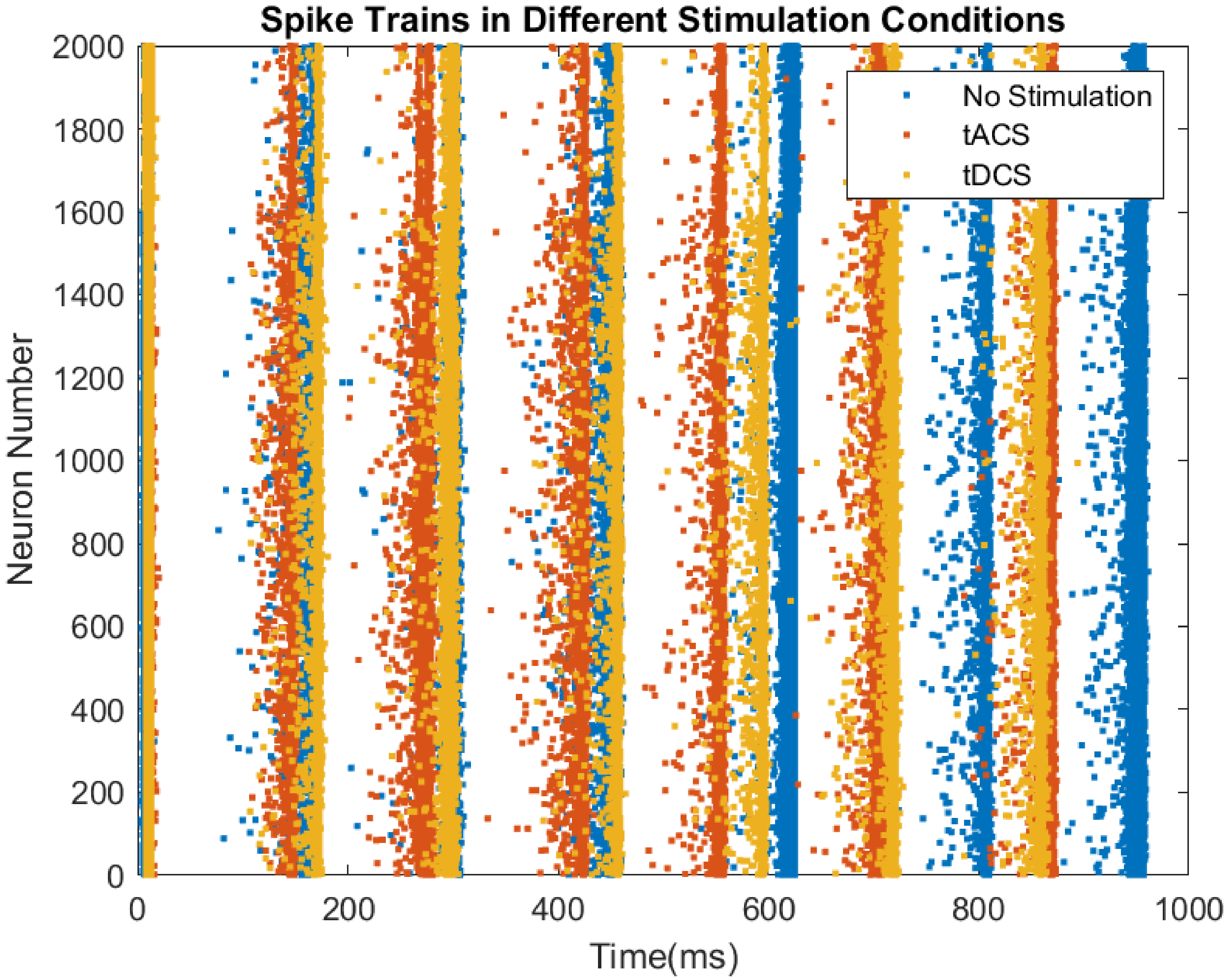
Spike Trains in Different Stimulation Conditions
No Stimulation
tACS
tDCS
Neuron Number
Time(ms)
0
200
400
600
800
1000
1200
1400
1600
1800
2000
0
200
400
600
800
1000

Figure S6

A

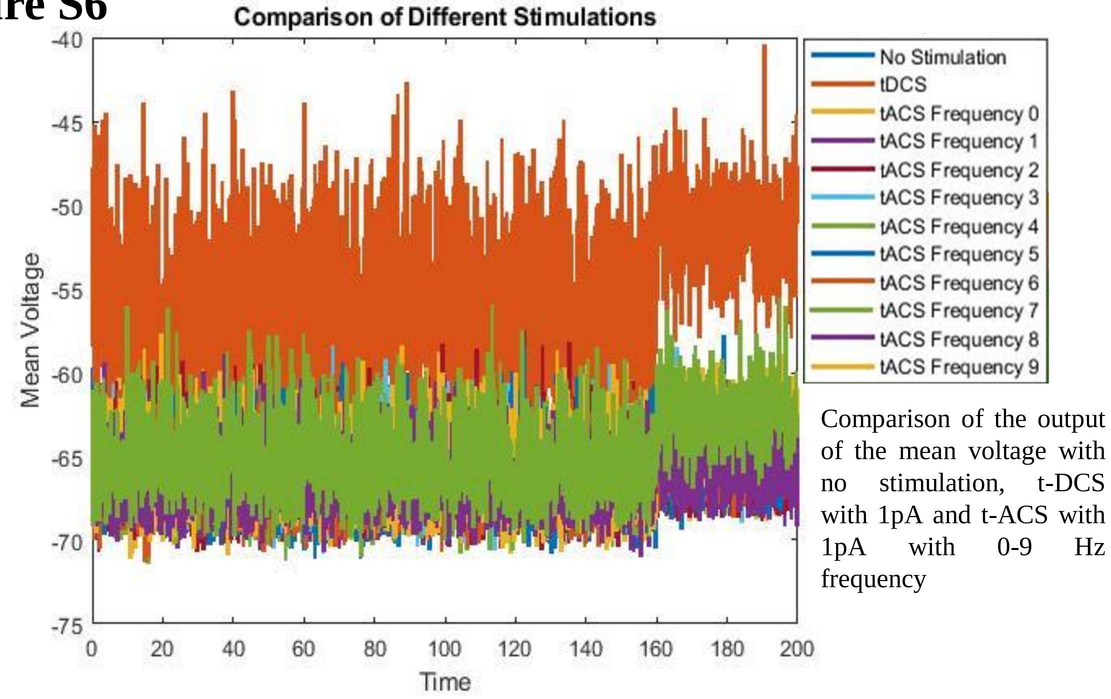

Comparison of the output of the mean voltage with no stimulation, t-DCS with 1pA and t-ACS with 1pA with 0-9 Hz frequency

B

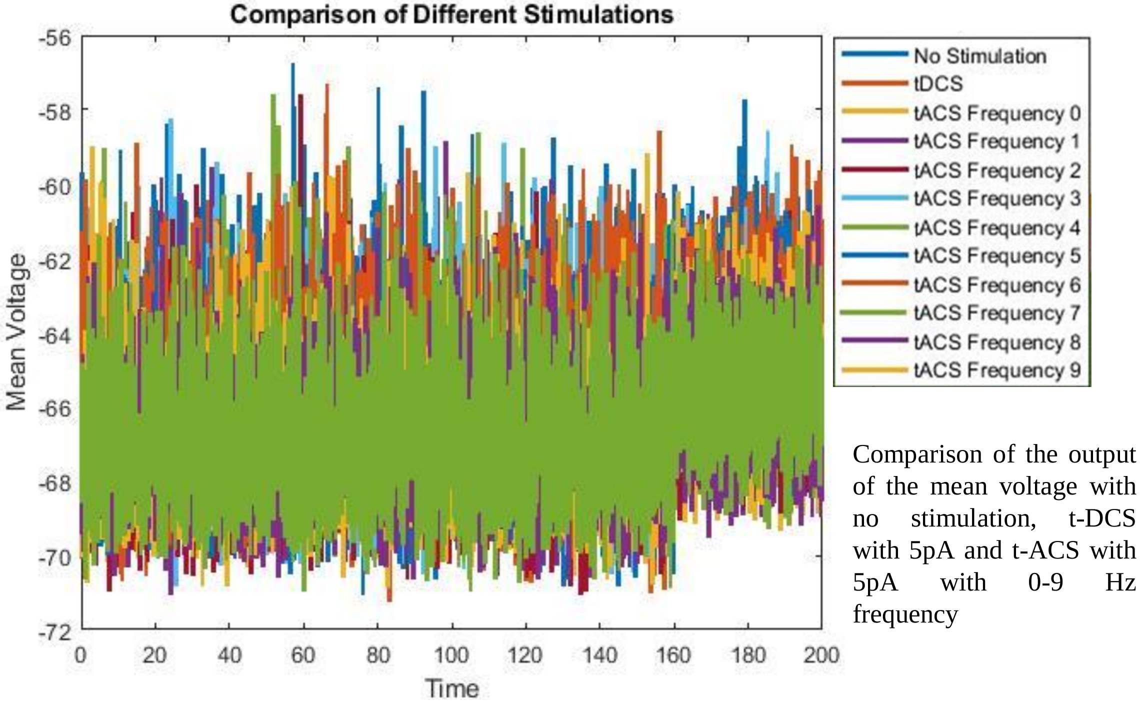

Comparison of the output of the mean voltage with no stimulation, t-DCS with 5pA and t-ACS with 5pA with 0-9 Hz frequency

C

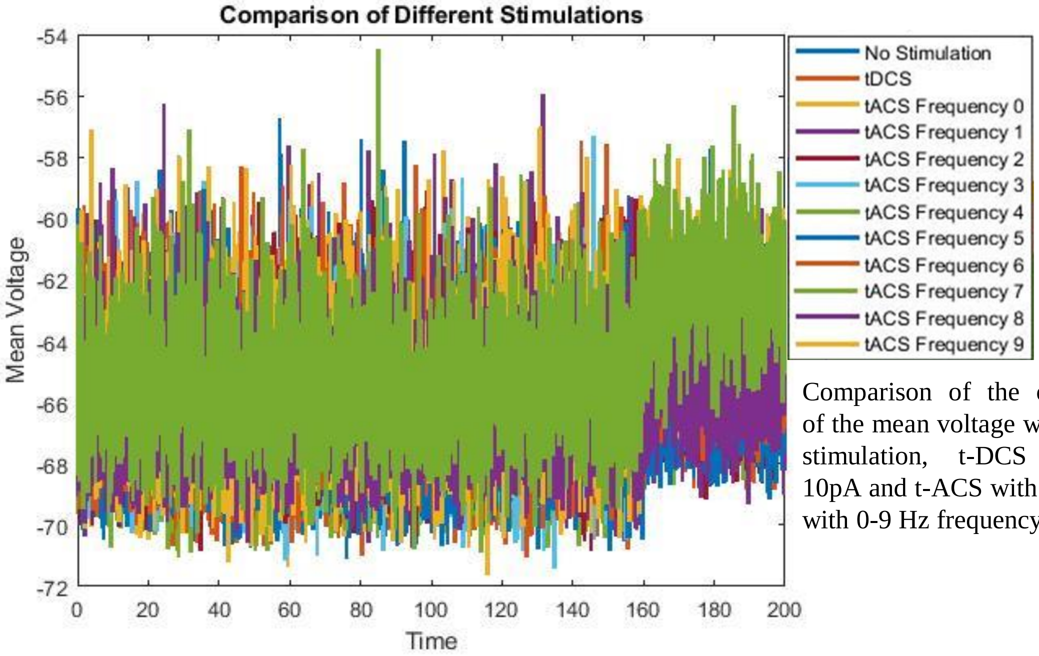

Comparison of the output of the mean voltage with no stimulation, t-DCS with 10pA and t-ACS with 10pA with 0-9 Hz frequency

D

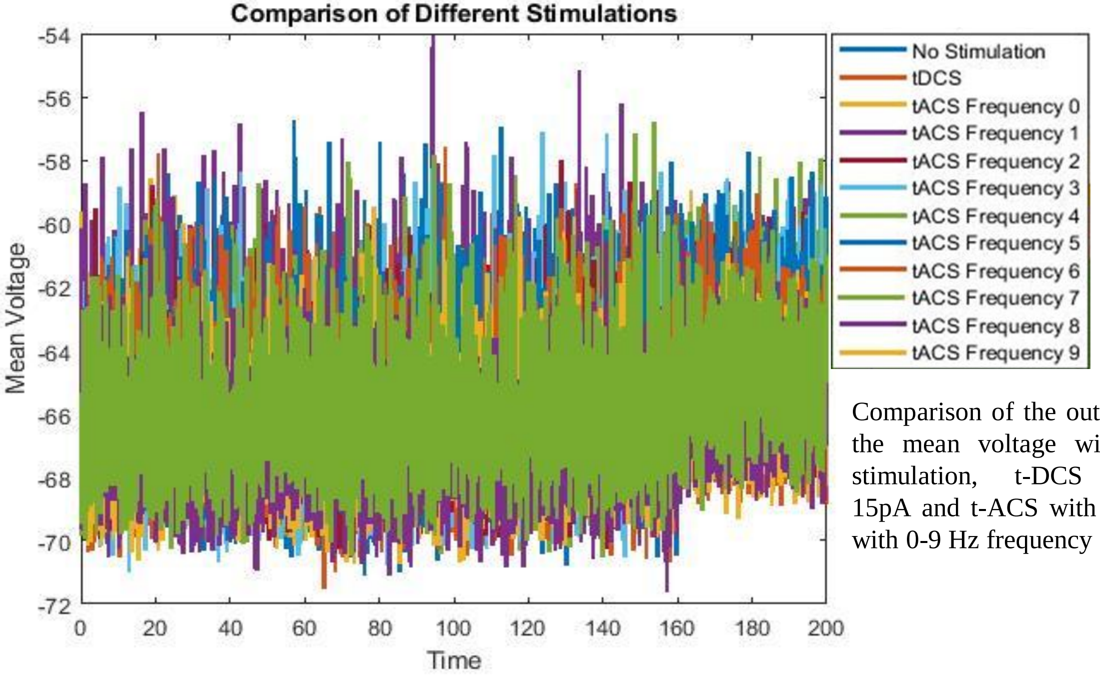

Comparison of the output of the mean voltage with no stimulation, t-DCS with 15pA and t-ACS with 15pA with 0-9 Hz frequency

Figure S7

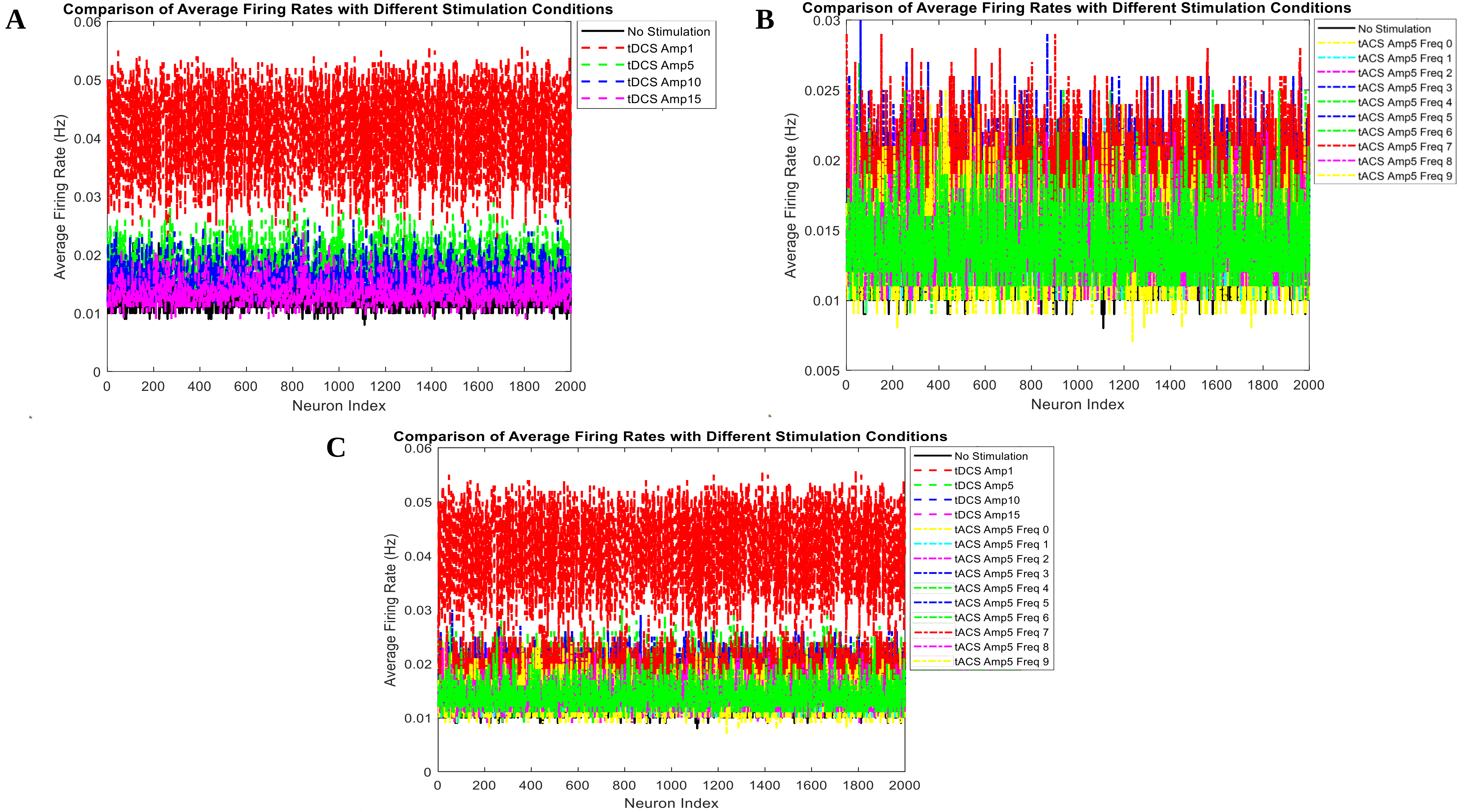
A
Comparison of Average Firing Rates with Different Stimulation Conditions
Average Firing Rate (Hz)
Neuron Index
No Stimulation
tDCS Amp1
tDCS Amp5
tDCS Amp10
tDCS Amp15
B
Comparison of Average Firing Rates with Different Stimulation Conditions
Average Firing Rate (Hz)
Neuron Index
No Stimulation
tACS Amp5 Freq 0
tACS Amp5 Freq 1
tACS Amp5 Freq 2
tACS Amp5 Freq 3
tACS Amp5 Freq 4
tACS Amp5 Freq 5
tACS Amp5 Freq 6
tACS Amp5 Freq 7
tACS Amp5 Freq 8
tACS Amp5 Freq 9
C
Comparison of Average Firing Rates with Different Stimulation Conditions
Average Firing Rate (Hz)
Neuron Index
No Stimulation
tDCS Amp1
tDCS Amp5
tDCS Amp10
tDCS Amp15
tACS Amp5 Freq 0
tACS Amp5 Freq 1
tACS Amp5 Freq 2
tACS Amp5 Freq 3
tACS Amp5 Freq 4
tACS Amp5 Freq 5
tACS Amp5 Freq 6
tACS Amp5 Freq 7
tACS Amp5 Freq 8
tACS Amp5 Freq 9
